# Ageing, sport and physical activity participation in Scotland

**DOI:** 10.3389/fspor.2023.1213924

**Published:** 2023-09-26

**Authors:** R. C. Richard Davison, Daryl T. Cowan

**Affiliations:** ^1^ Centre for Culture Sport and Events, University of the West of Scotland, Paisley, United Kingdom; ^2^ Sport and Physical Activity Research Institute, University of the West of Scotland, Paisley, United Kingdom

**Keywords:** sports participation, physical activity trends, older age groups, CMO guidelines, factors influencing participation rates

## Abstract

**Aim:**

As sport and physical activity are vital to support extended health spans, this study aimed to analyse the current trends in sports participation and physical activity rates among individuals aged 65 years and older in Scotland. Data were compared with the Chief Medical Officer (CMO) guidelines and analysed the influence of key factors on participation rates.

**Methods:**

The study used data from the Scottish Health Survey and the Scottish Household Survey (2019) to investigate self-reported participation in physical activity and sports across different age groups. Descriptive statistics and cross-tabulations were used to analyse the relationships between participation rates and influencing factors. Participation data for Parkrun events in Scotland were also analysed for the years 2008–2018.

**Results:**

The study found a clear decline in sports participation with age, with a steep decline after the age of 65, particularly in women. The majority of participation among individuals aged 65+ was in walking, with a sport participation rate of only 31.2% when walking was excluded. Physical activity and sport participation was lower in women across all age ranges but particularly so in the 75+ age group. The most popular sporting activities in the older age group were keep fit/aerobics, swimming and golf. Additionally, the study found that social deprivation had a major impact on sports participation rates, with the most deprived households exhibiting the lowest participation levels irrespective of age. The prevalence of loneliness was lower among individuals who participated in sports or adhered to the CMO guidelines for moderate/vigorous physical activity and strength-building exercises.

**Discussion:**

The findings of this study have implications for promoting physical activity and sports participation among older adults, particularly in deprived communities. This study highlights the importance of balance exercises within sport and the need for more targeted efforts to increase participation rates among older adults. The study also emphasizes the positive impact of sports participation on reducing loneliness among older adults. Overall, the findings suggest the need for ongoing efforts to promote physical activity and sports participation among older adults to improve their overall health and well-being.

## Introduction

**Figure 1:**
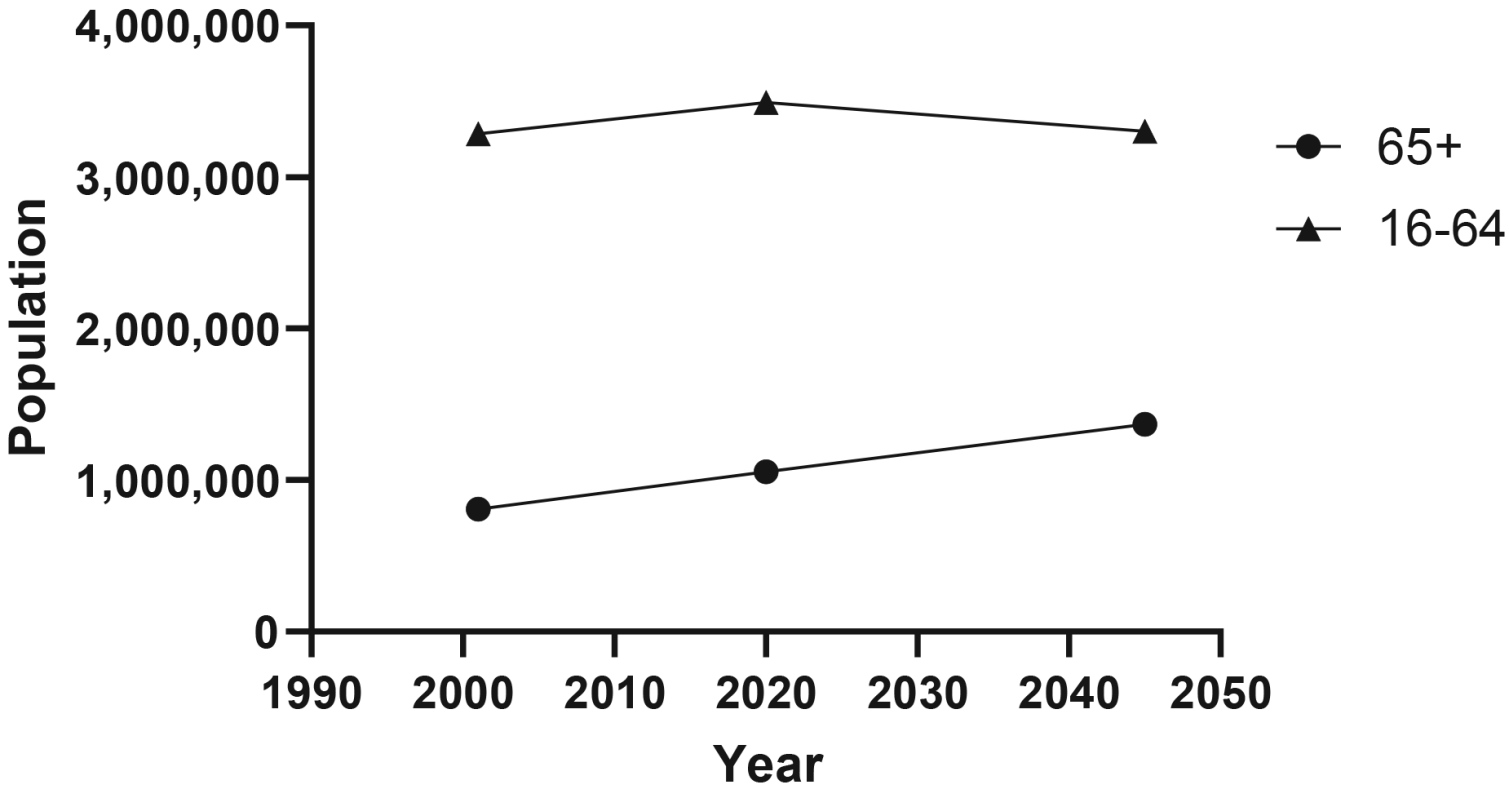
Population estimates and projections for Scotland (5).

Across most developed societies, because of reduced birth rates and improved healthcare, there is a demographic shift resulting in increasingly ageing populations. According to the Scottish Government, the population of those over the age of 65 increased from 807,000 in 2001 to 1.07 m in 2021 with a projected increase to just over 1.37 m in 2045 (30% increase), with the overall population of Scotland only increasing by 0.23 m over the same period and the population of working age declining by about 192,000 (Figure 1). The proportion of those 65+ in Scotland will grow from 19.4% to 25.5% of the total population by 2045. The projection to 2045 suggests a ∼65% increase in the over 75 age group. An additional effect is increasing life expectancy across most developed countries; this is projected to increase by 7.1 years (9.1%) for men and 6.4 years (7.7%) for women in the UK by 2050 (1). This clearly indicates a shift in the population towards older age groups with a number of social and physical/health consequences as the gap between life expectancy and health expectancy grows. The physical/health consequences typically associated with aging include, frailty, lung, breast and colon cancer, cardiovascular disease, metabolic syndrome, osteoporosis and osteopaenia, yet in many cases preventative strategies exist to delay these consequences and prolong health span (2). The social impacts are felt both for the individual in terms of their ability to contribute to society and more widely to general society, including significant economic impact of and caring for the older generations with multiple morbidities (3). Overall the likelihood of participation in sport, regardless of age, is multidimensional and participation can be predicted by the level of “sporting capital” which consists of physiological, social and psychological domains and is heavily influenced by socio-cultural norms (4).

The current Scotland Life Expectancy (LE) from birth (born 2019–2021) is in decline (male 76.6 years, female 80.8 years) and is the lowest in the UK (UK: male 79.0 years, female 82.9 years). While a recognised factor, COVID-19 has only had a minor impact decreasing life expectancy by 8–11 weeks. Current Healthy Life Expectancy (HLE) (male 60.4 years, female 61.1 years) is significantly below the UK average of (62.8 and 63.6 respectively) and has been in decline since 2014-16 (5). The LE and HLE place Scotland in the lower half of both statistics in Europe. At age 65, Scottish men have the lowest Disease Free Life Expectancy (DFLE) in the UK (8.6 years) (ONS). While many factors determine life expectancy, and in particular healthy life expectancy, physical activity is a key factor (5, 7) Community sport has the potential to make a significant contribution in increasing the physical activity levels of the older age groups improving and maintaining their physical, psychological and social health status.

However, there is a general trend of reduced participation in recreational sporting activities with age, starting as early as 12 years old and becoming even more pronounced in older age groups. The challenge in many countries, including Scotland, is understanding the reasons for the steep declines in participation with age and developing evidence-based policies to counter this trend of reduced participation. However, although much lower in overall magnitude, competitive masters sport is an exception to this trend, as it has seen a significant increase in popularity since the 1960's (8).

Scotland like most developed countries enjoys a relatively good quality of life, however it also has a wide spectrum of socioeconomic circumstances (SEC) and deprivation. There is some research on the impact of both early and later-life SEC on physical activity levels (9) and as might be expected, lower SEC does increase the risk of physical inactivity. There is no equivalent Scottish data on sport participation and SEC particulary in older age groups. The recent Observatory for Sport in Scotland (OSS) Academic Review paper, Sport and social inequality - unloading the dice (10) looks at the impact of inequality on sporting participation in the wider context, but the interaction of age and inequality on sport participation is much more difficult to determine. Although there are no direct data on this interaction, we do know that there are approximately 140,000 pensioners considered to be living in relative poverty (10) which will inevitably impact on sport participation in this age group. In Scotland, the Scottish Index of Multiple Deprivation (SIMD) is a geographic-based statistical tool used by local authorities, the Scottish government, the NHS, and other government bodies in Scotland, including national surveys, to identify areas with relatively high levels of deprivation.

Overall, sport is recognised as a significant contributor to physical health by promoting the accumulation of sufficient physical activity along with recognised social and psychological benefits. Moreover, sport is likely to provide both aerobic and strengthening exercise, which have a significant additive effect on reducing mortality (11). Physical inactivity has also been shown to increase with age more so in women and is higher in high - income countries (12).

The Chief Medical Officers (CMO) of the UK recommend that adults should aim to be active daily, achieving at least 150 min of moderate intensity physical activity per week. Specifically for older adults, they recommend that they should be as physically active as possible within their capabilities. They suggest that older adults should incorporate activities to improve balance and flexibility, and muscle-strengthening exercises, on at least two days a week. They further recommend that older adults should engage in aerobic activity at a moderate intensity level for at least 150 min per week or engage in 75 min of vigorous intensity aerobic activity. The aim of these recommendations is to promote healthy ageing and independence by reducing the risk of falls, improving cardiovascular health, maintaining cognitive function, and supporting social engagement (13).

This paper analyses the existing data to reveal the current sports participation rates and physical activity trends in the older age groups (65+) in Scotland. The data will be compared to the CMO guidelines on physical activity and with equivalent analyses in other countries around the world, and will also include the exploration of some of the key factors known to influence participation rates including sex and deprivation.

## Materials and methods

Using publically available data from the Scottish Health Survey (13) and Scottish Household Survey (14), we investigated the self-reported participation in sport and physical activity levels in Scotland across the age ranges to determine the differences with age and how this compared with the recommended amount of exercise for health as recommended by the Chief Medical Officers of the UK (15). In addition, we obtained participation data for Parkrun in Scotland. Parkrun is a free, weekly, timed 5-kilometer run that takes place in various parks and open spaces across the United Kingdom. We analysed data for the years 2008–2018 broken down by age group and sex with permission from the Parkrun Research Board. Approval from the local ethics committee was sought but determined as not required.

Where appropriate, we performed descriptive statistics on subgroups across the age groups and used cross tabulations to investigate the relationships between participation and the known factors influencing participation. We also compare our data to international data available in other academic publications and reports.

In Scotland, two of the main data sources used to measure sports participation are the Scottish Household Survey (SHS) and the Scottish Health Survey (SHeS). Both surveys feature high quality methodologies, however the questions regarding sporting participation are rather limited, resulting in a lack of depth of information available. In addition, both surveys are carried out all year round but do not document the date of collection, and in the case of sport and physical activity participation, there is a well-documented seasonal variation with individuals being more active in the summer compared to the winter (16).

The physical activity section of the Scottish Health Survey has a number of specific questions on participation in “Sport and Exercise”. Respondents can choose from a list of 40 sports and exercises that they may have participated in over the last 4 weeks. This includes details on asking the frequency, duration and intensity of participation in that time frame. As the SHeS includes questions on walking and other recognised physical activities such as heavy housework, heavy manual/gardening/DIY, these activities can be separated from sports participation and thus give a more detailed description of overall physical activity. The SHS consists of two simple questions, what activities (from a list of 12 including “other” option) have you taken part in the last four weeks, followed by how many days in the last four weeks did you do at least one of the activities. Given the varying methodologies, it is understandable that the data from these two surveys yield somewhat different overall outcomes.

We defined older people as those aged 65 years and older. However, we occasionally refer to data and research from slightly younger (50–65) cohorts where significant information indicates important trends or concepts.

## Results

**Figure 2:**
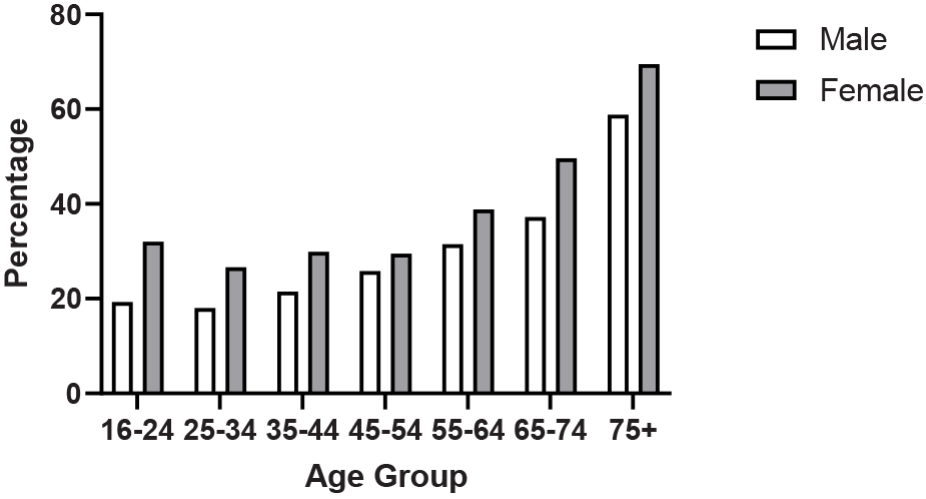
Percentage of adults not meeting the CMO recommendations for moderate/vigorous physical activity and strengthening recommendations (13).

Figure 2 shows a clear increasing percentage of individuals not meeting the Chief Medical Officers (CMO) of the UK (15) guidelines for activity duration and strengthening exercise from both physical activity and sport participation from age 25. This is particularly marked for both male (58.9%) and females (69.5%) over the age of 75 years, significantly more than the overall population average of 35.5%.

Balance exercise is a key component of the CMO physical activity recommendations and undertaking balancing exercise was significantly related to sport participation in the SHeS 2019 data. Of those over the age of 65 who did not take part in sport, 85.4% had not undertaken any balance improving activity in the previous 4 weeks. This contrasts with 56.9% of those who did participate in sport undertaking 2 or more balance improving activities per week in the previous 4 weeks.

**Figure 3:**
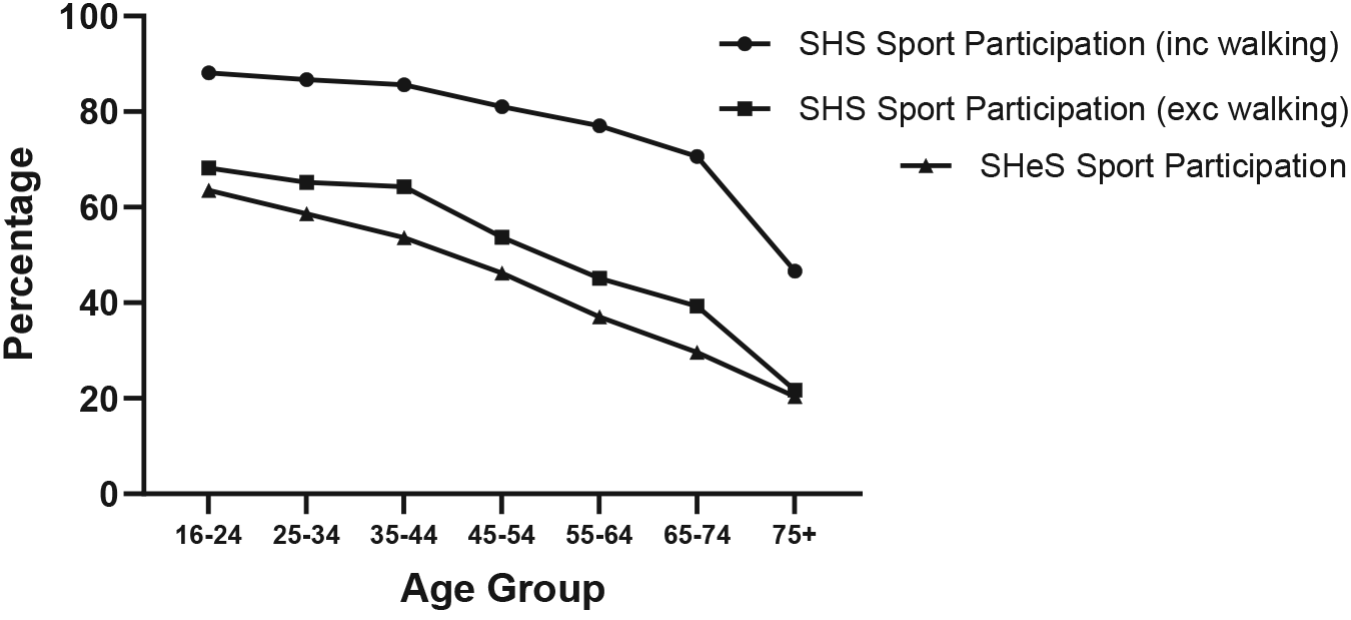
Sport participation levels (including and excluding walking) from both the (13, 14).

Both the Scottish Health Survey and the Scottish Household Survey show a clear decline in sport participation with age (Figure 3), defined as taking part in a particular sport in the last 4 weeks. The exclusion of walking significantly decreases absolute values by an average of 52%, but the pattern remains similar with a steeper decline after the age of 65. In the Scottish Household Survey 59.5% of adults aged 65+ participated in sport (including walking), in the last 4 weeks (14), which contrasts to a participation rate of 82.8% for those 16–64. However, the majority of that participation is accounted for by walking as participation for 65+ drops to 31.2% when walking is removed. The data from the SHeS are consistently lower with a participation rate for 65+ of 25.6%.

When asked about sporting activity over the previous 4 weeks in the Scottish Household Survey, the number one activity for the population as a whole was walking (at least 30 min for recreational purposes) this was replicated in the older age group, although the participation rate within the age group was significantly lower for those over 65 (71.6% vs. 51.3%). Keep fit/aerobics, swimming and golf were also popular with the 65+ age group.

**Figure 4:**
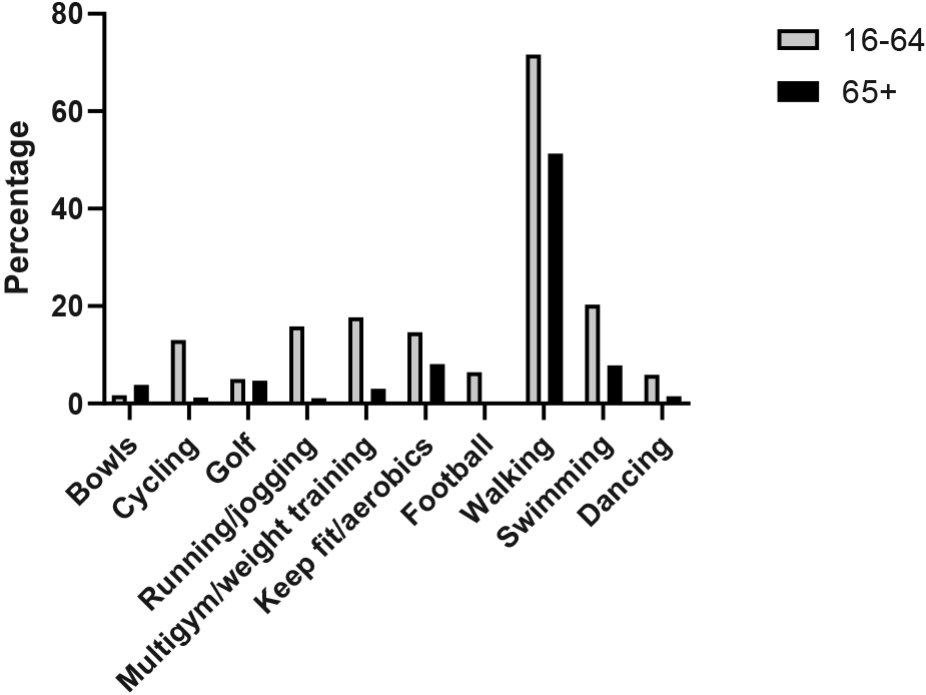
Top ten sports with participation rates over the previous 4 weeks within adults aged under and over 65 years (SHS 2019).

Across every activity, participation was lower in absolute terms in the older age group except for bowls where participation was higher in the 65+ age group (3.8% vs. 1.7%) (Figure 4). The sport with the next closest participation rates was golf with equivalent older (4.7%) and younger (5%) participation rates.

**Figure 5:**
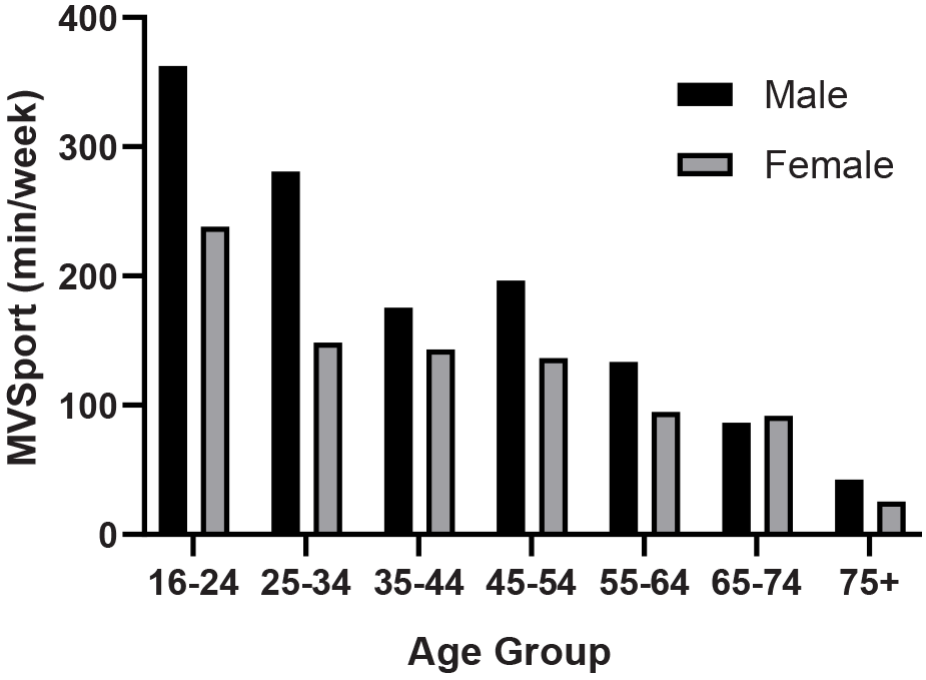
Sex differences in moderate and vigorous physical activity from sport for different age groups (SHeS 2019).

Analysing the total time of sport participation per week at either moderate or vigorous intensity demonstrates a significant decline in overall sporting activity with age although somewhat maintained from ∼25–55, particularly for women, there are much more marked declines in later age (Figure 5). From the 16–24 age group peak of 317.5–445.1 min (95% CI), there is a decline to 50.1–77.4 min (95% CI) in the 75+ age group. Overall, there is an 88.3% decline for men and an 89.3% decline for women from 16 to 24 to 75+ age. As would be expected, there is a significantly lower volume of activity for women except for the 65–74 age group, where the activity volume is better maintained for women compared to men. However, the sex differential is reestablished in the 75+ age group.

Data from participation in Parkrun events in Scotland show a near exponential growth in numbers over the 10 year period 2008–2018 for both men and women. However, this should be set in context with the overall growth of parkrun in all age groups over the same period. Nevertheless, the proportion of those from the 65+ age group taking part has risen from 1% to 5% for men and from 0% to 3% for women. However this data also shows a very large sex difference with 3.5 times more men taking part.

**Figure 6:**
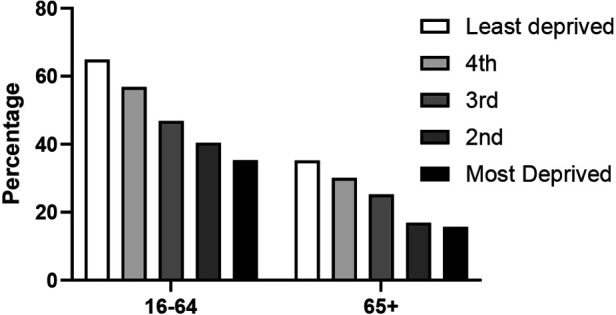
The interaction of SIMD and age on sport participation (SHeS 2019).

Social deprivation, classified by the Scottish Index of Multiple Deprivation, has a major impact on sport participation with the most deprived households exhibiting the lowest sport participation levels with overall sport participation of only 30% regardless of age. Figure 6 demonstrates the impact of deprivation across the quintiles for the 65+ age group, showing a decline in participation across the quintiles, which is equivalent to the younger age groups, to a participation rate of 15.7% for the most deprived.

**Figure 7:**
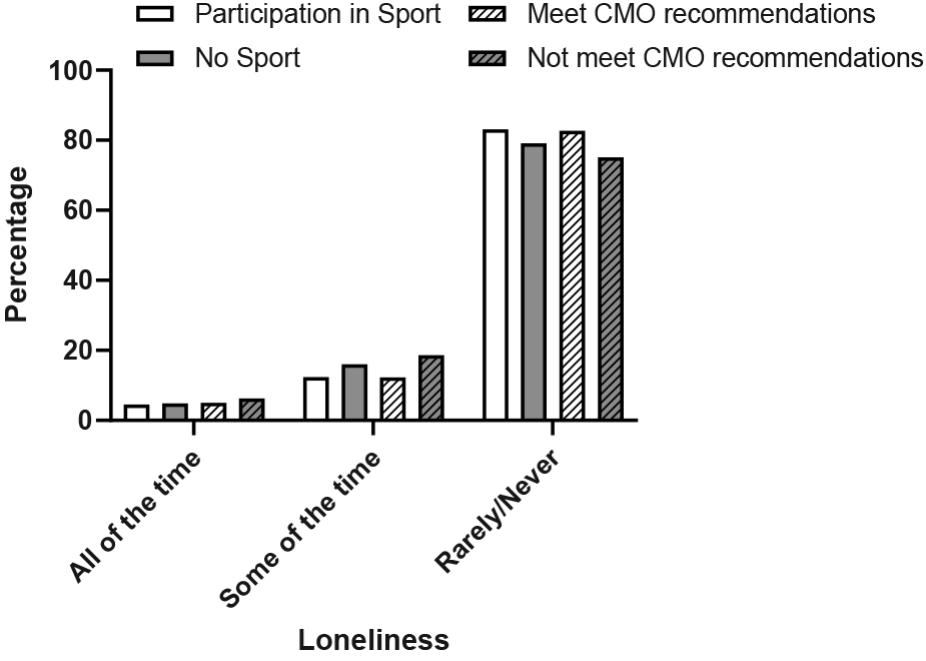
Relationship between loneliness in the last two weeks and sport participation in the last 4 weeks and meeting the CMO recommendations for MVPA and strengthening exercises for individuals over 65 years of age. (SHeS 2019).

In general, the prevalence of loneliness was reduced among individuals engaging in sports or adhering to the CMO guidelines for MVPA and strength-building exercises (Figure 7). This observation was particularly pronounced in instances of occasional feelings of loneliness, where the percentage of participants reporting such experiences was 22.5% and 33.9% lower, respectively.

The top 5 reasons for taking part in activity were similar between the younger and older age groups, but the rank order of the reasons did differ. While enjoyment and keeping fit were the top two for both groups the enjoyment was a higher priority for the older group, who also ranked socialisation much higher than younger individuals.

## Discussion

Like many countries, Scotland has a significantly increasing population of older adults, which is projected to continue to 2050 and beyond (Figure 1). This demographic shift poses significant challenges to healthcare services, with the underlying causes being intricate and multifaceted, necessitating a comprehensive public health approach (17). Scotland's life expectancy, particularly healthy life expectancy, is currently the lowest in the UK and experiencing a decline. Consequently, it is imperative for policymakers to devise strategic plans aimed at promoting healthy ageing in order to prolong the duration of healthy life expectancy and significantly reduce the potential health care cost burden (18). Noncommunicable diseases (NCDs) serve as primary contributors to mortality and disability among elderly individuals (19), further exacerbated by low levels of physical activity (5). As such, attaining a thorough understanding of factors influencing physical activity and sports engagement among older age groups is crucial for developing effective strategies that encourage active lifestyles within this demographic. Thus, with the available data, this paper aimed to describe the current situation in Scotland as a potential baseline for key stakeholders and policy makers.

A challenge in interpreting research literature in this field is the conflation of sports activities with the more general term, “physical activity”, which becomes notably complex in older demographics where competitive sport participation is less common. In this context, we endeavour to distinguish between sport and other forms of physical activities, primarily focusing on sport participation. A sports participant can be defined as an individual directly or indirectly involved in sports, including roles such as player, contestant, team member, coach, manager, trainer, or administrator. It is widely recognized that the majority of physiological, psychological, and social benefits are derived from physically participating as a player, contestant, or team member. However, non-physical roles such as coach, manager, trainer, or administrator have also been shown to provide documented psychological and social advantages (20). Although there is a lack of specific data on this subject, it is reasonable to assume that such involvement and social engagement could result in increased physical activity levels and subsequently promote health benefits (21). For many people, sport participation constitutes their primary source of weekly physical activity. However, inconsistency exists across age groups; younger adults are more likely to gain their weekly physical activity through sport than those over 55 (22). For the purposes of this paper, we focus on physical sports participation, only referring to the broader data on physical activity where a clear parallel can be drawn and there is a lack of distinct or equivalent research data on sports participation. We defined older people as those aged 65 years and older. However, we occasionally refer to data and research from slightly younger (50–65) cohorts where significant information indicates important trends or concepts.

Adequate overall physical activity levels are critical for the maintenance of good health (11) and there are several standards throughout the world, that broadly relate to a similar acceptable threshold of physical activity. Across Europe (23, 24) and most other countries (25) and in Scotland, it is clear that both general physical activity and specific sports participation declines with age Figure 3.

The data concerning Scotland's population demonstrate a considerable decrease in sufficient physical activity as individuals age, with 43.7% of those aged 65–74 and 65% of those aged 75 and above not adhering to the Chief Medical Officers' recommendations for either moderate to vigorous physical activity (MVPA) or strength training. One could argue that imposing a uniform physical activity target for all age groups may be impractical and that tailored guidelines for specific age ranges are warranted. While the latest “UK Chief Medical Officers” Physical Activity Guidelines' do acknowledge the 65+ demographic as distinct, the MVPA criteria remain unchanged compared to younger adults (15). Of further concern is the substantial proportion of elderly individuals, especially women, who do not fulfill either the MVPA or strength training recommendations, with nearly 70% of women in the 75+ age bracket falling short of these guidelines. Considering that the recognised physiological consequences of aging are accelerated beyond the age of 65 even for those who undertake PA (26, 27), this is even more accelerated for those who do not exercise or stop exercise (28).

In older age balance, coordination and strength are key factors for the risk of falling (29), and exercise has repeatedly been shown as an effective intervention to reduce fall rate (30, 31); thus, all three are included in the CMO Physical Activity Guidelines. Many sports have the advantage of contribution to training of all three factors and sport participation of as little as at least once a week has been shown to reduce the likelihood of falls by more than 20% (32). Within the Scottish Health Survey, it is important to recognise that the measure of balance improving exercise is largely derived from involvement in certain sports, but it is not exclusively so, it is also derived from activities backed up with additional follow-up questions related to getting up an moving around as part of that activity. Thus it is not surprising that we found a strong relationship between sport participation generally and meeting the CMO recommendations for balance improving activities at least twice a week. However, the fact that 85.4% of those who indicated no specific sport participation did not undertake any balance improving exercise, or at least it was not identified from the methodology used in the survey, suggests that these individuals would therefore be at an additional risk of falls. This is supported by Gale et al. (33) who reported an odds ratio 2.57 (95% CI: 1.42, 4.66) for falls for those older individuals who had a sedentary lifestyle compared to the most active quintile (33).

Walking is not considered a sport but clearly in Figure 3, walking is a key component of overall regular physical activity for all ages and may be more important for older individuals who generally do not participate in organised sport. Sport can make a significant contribution to the overall levels of physical activity, particularly in younger age groups (22). Data from the SHeS (2019) in Figure 4 show that 77.4% of all physical activity in the 16–25 age group comes from sport. It has been reported elsewhere that the contribution of sport to overall physical activity declines with age (22), as is the case in Scotland with only a contribution between 25.6% and 31.3% to total physical activity from sport.

The elevated numbers of sports and exercise participation in the SHS compared to the SHeS, as seen in Figure 3, can be primarily attributed to the slight variation in question construction, leading to changes in the data sources used for calculations. A significant element influencing the stated participation percentages is the inclusion or exclusion of walking as an activity. In the most recent pre-pandemic SHS (2019) data, adult participation in sports and exercise, including walking, stood at 76.5%, while excluding walking reduced it to 51.1% (SHS, 2019). For those aged 65 and above, eliminating walking from the data considerably decreases overall participation from 59.5% to just 25.6%. The SHS data without walking, presented in Figure 3, aligns closely with SHeS data and therefore likely offers a truer representation of the decline in sports engagement as people grow older. Equivalent data from Denmark shows no decline in “Sport and Exercise” with age, with the highest participation group being the 60–69 age group. More in-depth analysis of the number of times and hours of sport and exercise participation per week still confirms that the 60+ age group in Denmark are very active and at least as active as their younger counterparts (34).

**Figure 8:**
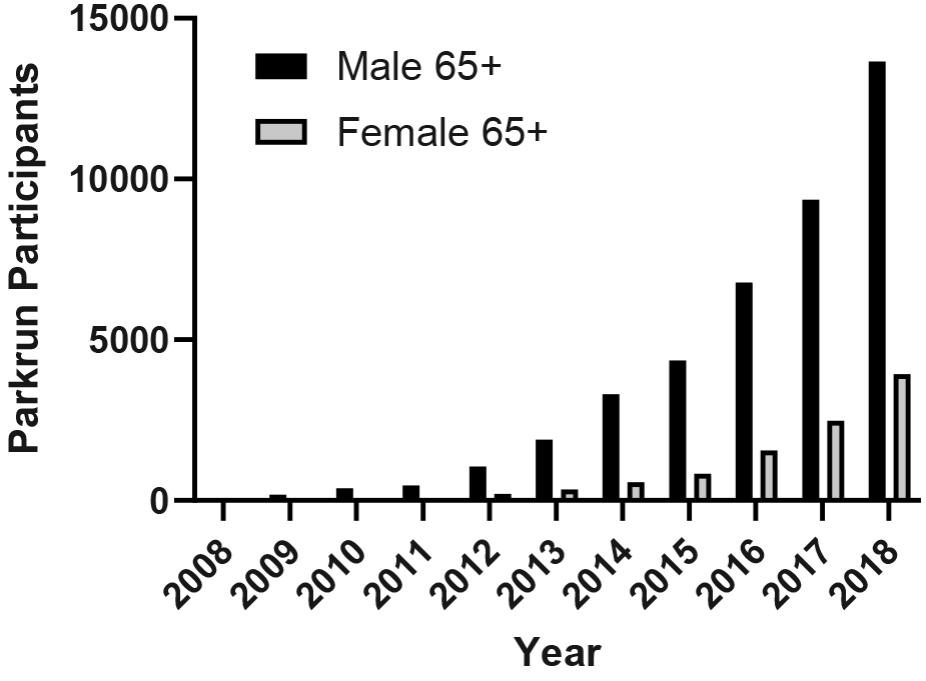
Participant numbers aged 65+ for Parkrun in Scotland (2008–2018).

In both men and women, the highest participation rates are found in the 16–25 age group, with a significant decline following this age range, resulting in around 60% lower participation in the 65+ age group. This figure is slightly higher than other cross-sectional data reported, which shows a 50%–60% difference (35–37). Numerous cross-sectional studies have documented trends over time and variations between age groups; however, there are very few longitudinal studies examining a cohort's participation over an extended period. Breuer and colleagues analysed participation rates spanning two decades, revealing only slight decreases in participation rates among those who were middle-aged at the beginning of the study. Nevertheless, it is essential to consider that during the study period (1985–2005), there was a significant increase in overall cohort participation rates, particularly among older age groups—a trend confirmed by several other research studies (35–37), as well as data on Park Run participation shown in Figure 8.

In Scotland and internationally, although there are a number of repeated cross-sectional surveys on sports participation across the age ranges, there is a distinct lack of longitudinal or cohort data. Cross-sectional studies cannot measure developments or differentiate between age and cohort effects as they are amalgamated within the data. Longitudinal approaches can measure development processes and monitor cohort effects, but age and period effects are combined and thus cannot be differentiated. A cohort sequence analysis period and cohort effects can be monitored; this is where the same population is measured at several time points with a new “youngest age group” added at every measurement point (35). The data on the impact of ageing on physical performance or physiological function show that longitudinal declines with age tend to be significantly larger than those measured from cross-sectional studies (38). However, a recent study harmonised the data from eight cohort studies assessing the impact of physical activity on healthy ageing trajectories and concluded that inactivity represented a 2.27–5.56 times increased odds of a poorer health trajectory (39). Additional longitudinal studies are also necessary to understand individuals' experiences as they enter advanced ages and confront challenges associated with maintaining their active lifestyle.

Across all age groups and most sports, men tend to participate more than women, a trend observed in Europe (24) and Australia. In England, female sports participation has seen a slight increase from 2008–9 to 2013–14, after controlling for other factors (40). Despite this growth, older women still generally participate in sports less frequently than older men. We demonstrated a significantly lower participation time for women compared to men in Figure 5 by examining the total weekly participation time at moderate or vigorous intensity. This finding is also supported by other data sources such as Parkrun (Figure 6), which indicates a significant increase in older women's participation over the last decade. Non-competitive sports tend to be more popular among older age groups, especially for women. A New Zealand study reveals that gender differences do not exist in non-competitive sports participation and that adult women actually participate more than men. However, these trends reverse when examining competitive sports (25). Unfortunately, we do not have access to data that examines the relationship between gender and competitive/non-competitive sports participation in Scotland.

Although there are limited direct measures of loneliness or social isolation in Scotland, the Scottish Health Survey does report on “Social Contact.” It shows that 15% of individuals over 65 have interactions with neighbors, family, and friends fewer than once or twice a week (SHeS 2019). Notably, this percentage is not much lower than that of people under 65 (13.6%). Men above 50 years old appear more susceptible to social isolation than women (41). While causality cannot be definitively established, Figure 7 implies that those participating in sports and meeting the CMO recommendations for MVPA and strengthening exercises are less likely to feel lonely. It seems logical that engaging in sports and physical activity could offer excellent opportunities to reduce loneliness and social isolation. However, baseline data and verified methods are scarce. Numerous studies of differing quality have investigated various interventions, including some involving sports and physical activity, with mixed results (42, 43). Limited research suggests that involvement in physical activities, including sports, can significantly improve feelings of loneliness and social isolation (43, 44). Additionally, participating in external social activities such as sport may reduce loneliness irrespective of socioeconomic status (45). The recent NHS Health Scotland report on “Social Isolation and Loneliness” emphasized the need for an agreed set of indicators leading to survey data to better understand the issue's scope and assess potential interventions (41).

Effective promotion of exercise and sport participation requires a tailored approach, as research indicates that age influences the motivation to exercise (41, 42). Table 1 of this study confirms previous findings that socialisation is a more significant motivator for physical activity among older populations (41, 43, 44). Interestingly, “to improve health” ranked lower in both age groups, whereas in other countries, it ranked higher (47). Despite the potential for common themes in the findings of various countries or regions, country-specific social norms and opportunities for participation are likely to impact the reported motivations (45). In the 65+ age group, “enjoyment” of exercise, or has been described in other research the “positive energy” of the exercise (41) or “pleasure” (51), was the top motivator, followed by the aim to “keep fit.” These motivators are crucial for promoting appropriate physical activity opportunities for older adults, as maintenance of function is highly motivational (41).

**Table 1:** Top 5 reasons to take part in activity in older and younger adults (SHeS 2019).

Reasons	16–64	65+
To keep fit (not just to lose weight)	1	2
To socialise	5	3
Just enjoy it	2	1
For health reasons/to improve health	4	4
To de-stress, relax and unwind	3	5

In conclusion, Scotland is experiencing a significant increase in its aging population, which poses significant challenges to healthcare services. Maintaining adequate physical activity levels is critical for good health, especially among older adults, who are more prone to non-communicable diseases and physical limitations. While sport participation can be an essential source of physical activity, its contribution decreases with age, particularly for women. Socialization was found to be a more significant motivator for physical activity among older populations, making it a crucial factor to consider when promoting exercise and sport participation in older age groups. The promotion of active lifestyles among older adults requires a tailored approach that considers country-specific social norms and participation opportunities. The findings of this study provide a baseline of the current situation in Scotland, which can be used by policymakers and key stakeholders to develop effective strategies to promote healthy aging, prolong healthy life expectancy, and reduce potential healthcare costs. Future research, particularly longitudinal studies, is necessary to understand the impact of aging on physical activity and to encourage active lifestyles among older adults in Scotland.

## Data Availability

Publicly available datasets were analyzed in this study. This data can be found here: https://doi.org/10.5255/UKDA-SN-8737-1.
